# Comparison of high throughput RNA sequences between *Babesia bigemina* and *Babesia bovis* revealed consistent differential gene expression that is required for the *Babesia* life cycle in the vertebrate and invertebrate hosts

**DOI:** 10.3389/fcimb.2022.1093338

**Published:** 2022-12-19

**Authors:** Janaina Capelli-Peixoto, Perot Saelao, Wendell C. Johnson, Lowell Kappmeyer, Kathryn E. Reif, Hayley E. Masterson, Naomi S. Taus, Carlos E. Suarez, Kelly A. Brayton, Massaro W. Ueti

**Affiliations:** ^1^ Program in Vector-Borne Diseases, Department of Veterinary Microbiology and Pathology, College of Veterinary Medicine, Washington State University, Pullman, WA, United States; ^2^ Veterinary Pest Genetic Research Unit, USDA-ARS, Kerrville, TX, United States; ^3^ Animal Disease Research Unit, USDA-ARS, Pullman, WA, United States

**Keywords:** Bovine babesiosis, *B. bigemina*, *B. bovis*, RNA-seq, blood-stages, kinetes, differential gene expression

## Abstract

Bovine babesiosis caused by *Babesia bigemina* and *Babesia bovis* is an economically important disease that affects cattle worldwide. Both *B. bigemina* and *B. bovis* are transovarially transmitted by *Rhipicephalus* ticks. However, little is known regarding parasite gene expression during infection of the tick vector or mammalian host, which has limited the development of effective control strategies to alleviate the losses to the cattle industry. To understand *Babesia* gene regulation during tick and mammalian host infection, we performed high throughput RNA-sequencing using samples collected from calves and *Rhipicephalus microplus* ticks infected with *B. bigemina*. We evaluated gene expression between *B. bigemina* blood-stages and kinetes and compared them with previous *B. bovis* RNA-seq data. The results revealed similar patterns of gene regulation between these two tick-borne transovarially transmitted *Babesia* parasites. Like *B. bovis*, the transcription of several *B. bigemina* genes in kinetes exceeded a 1,000-fold change while a few of these genes had a >20,000-fold increase. To identify genes that may have important roles in *B. bigemina* and *B. bovis* transovarial transmission, we searched for genes upregulated in *B. bigemina* kinetes in the genomic datasets of *B. bovis* and non-transovarially transmitted parasites, *Theileria* spp. and *Babesia microti*. Using this approach, we identify genes that may be potential markers for transovarial transmission by *B. bigemina* and *B. bovis*. The findings presented herein demonstrate common *Babesia* genes linked to infection of the vector or mammalian host and may contribute to elucidating strategies used by the parasite to complete their life cycle.

## Introduction

Bovine babesiosis is a tick-borne disease of worldwide impact caused by Apicomplexan intracellular parasites of the genus *Babesia*. The four species of *Babesia* responsible for most of the clinical cases in cattle are *Babesia bigemina*, *Babesia bovis*, *Babesia divergens*, and *Babesia major* (Bock et al., 2004). The first two are found in several countries in tropical and subtropical regions, including Central and South America, Australia, Asia, Africa, and Southern Europe with high prevalence of 22% for *B. bigemina* and 20% for *B. bovis* (Jacob et al., 2020).

The severity of bovine babesiosis can vary according to several factors, including age, genetics, and status of the immune system, among other characteristics, including the relative virulence of the *Babesia* strains involved (Bock et al., 2004; Schnittger et al., 2012). The most common manifestations of acute cases include fever, hemolytic anemia, jaundice, weakness, and hemoglobinuria while chronic infections are usually asymptomatic (Bock et al., 2004; Schnittger et al., 2012). Another symptom of acute *B. bovis* infection is the manifestation of neurological signs (ataxia, lethargy, inappetence, incoordination, teeth grinding, involuntary movements of the legs) which are related to sequestration of infected erythrocytes in capillaries of different tissues, including the brain. However, this manifestation does not happen during *B. bigemina* infections (reviewed by (Gallego-Lopez et al., 2019). In acute infections, the maximum percentage of infected erythrocytes is ~10% in *B. bigemina* and less than 1% in *B. bovis* (Chaudhry et al., 2010), but these estimates may be biased by massive sequestration of *B. bovis* infected erythrocytes in capillaries. Disease triggered by *B. bigemina* infection causes mortality rates of up to 30% in untreated animals, whereas infections by *B. bovis* may have much higher mortality rates (between 70-80%) (Jacob et al., 2020).

Both *B. bigemina* and *B. bovis* are transmitted to cattle by *Rhipicephalus* ticks (Bock et al., 2004), the definitive host of *Babesia*. The vertebrate host infection starts when sporozoites are injected along with tick saliva by the infected nymph (*B. bigemina*) or larva (*B. bovis*) (Suarez and Noh, 2011). Invasion occurs after several random collisions between parasite and erythrocytes which facilitates orientation of the anterior *Babesia* apical complex. After a tight junction between sporozoites and the erythrocyte cell membrane is established, proteins are released from the secretory organelles, and the sporozoite actively penetrates the erythrocyte. Internalized parasites differentiate into trophozoites which reproduce asexually into merozoites. Mature merozoites egress from the infected erythrocyte, causing their destruction, and invade other erythrocytes, where the cycle of asexual multiplication is perpetuated (Mosqueda et al., 2002; Jalovecka et al., 2018). If the animal survives acute disease, they become chronically infected for months to years (Johnston et al., 1978; Chung et al., 2017). When ticks ingest the parasites during blood meal acquisition, gamogony begins with the formation of gametocytes in the tick midgut lumen. Gametocytes undergo metamorphosis resulting in haploid gamete formation (Jalovecka et al., 2018). *Babesia* fertilization is induced by contact between male and female gametes and results in the formation of a diploid zygote, which invades tick gut epithelial cells. Inside the intestinal cells, zygotes undergo meiotic division resulting in the formation of a single cell kinete. Kinetes are released to the tick hemolymph and migrate throughout the whole tick body, including ovaries which results in vertical transmission to the tick offspring. In the infected larvae, *Babesia* infects the salivary glands and undergoes sporogony to become sporozoites. These infectious forms are continuously released to the blood of the vertebrate host during larval and nymphal feeding (Jalovecka et al., 2018).

All these *Babesia* stages and transitions are required to complete the parasite life cycle and are characterized by specific gene expression of proteins involved in distinct cell biological processes and molecular mechanisms, with different signaling pathways. However, our current knowledge regarding these events is limited (Jalovecka et al., 2018; Suarez et al., 2019; Ueti et al., 2020; Ueti et al., 2021). This knowledge gap precludes the understanding of host-tick-*Babesia* interaction, constraining the discovery of novel drug therapies and identification of new targets for vaccine development (Suarez et al., 2019).

In this study, we identified differentially expressed genes in both *B. bigemina* kinetes and blood-stages and compared our results with a recently published *B. bovis* transcriptome data set (Ueti et al., 2020; Ueti et al., 2021). The findings presented herein demonstrate common *Babesia* genes linked to tick vector and mammalian host infection that may contribute to elucidating strategies used by the parasite to complete their biological life cycle.

## Materials and methods

### Ethics statement

All animal procedures were carried out in accordance with the Animal Welfare Act and Regulations (United States Department of Agriculture). The study was conducted under Animal use protocol #IACUC 2018-16, approved by the Institutional Animal Care and Use Committee of the University of Idaho, Moscow, ID, USA and adhered to the principles and standards put forth in the Guide for the Care and Use of Laboratory Animals (The National Academy of Sciences) and the Guide for Care and Use of Agricultural Animals in Agricultural Research and Teaching (The American Dairy Science Association, the American Society of Animal Science, and the Poultry Science Association).

### Cattle, *Rhipicephalus microplus* and *Babesia bigemina*

Three splenectomized naïve Holstein calves (C46527, C1437 and C46541) approximately 4 months old and confirmed to be *Babesia*-free by competitive enzyme-linked immunosorbent assay and nested PCR (Goff et al., 2006; Suarez et al., 2012) were used for feeding *R. microplus*, the La Minita strain. Approximately 40,000 larvae were applied under a cloth patch on the dorsal region for acquisition of *B. bigemina* parasites. Fourteen days after tick application, when approximately 1% of the ticks had molted to unfed adults, ~10^7^ *B. bigemina* infected erythrocytes, the Mexico strain, were inoculated intravenously into each calf to synchronize female tick acquisition feeding with an ascending *B. bigemina* parasitemia (Bohaliga et al., 2019). The presence of *B. bigemina* in the blood was determined by Giemsa-stained blood smear.

### Isolation of *Babesia bigemina* blood-stages and kinetes

For obtaining *B. bigemina* blood-stages, blood of each infected calf was collected individually immediately before euthanasia in sterile flasks with glass beads and shaken to prevent coagulation. Defibrinated red blood cells (RBCs) were washed with Puck’s saline G solution, placed in culture flasks, and incubated with 5% CO_2_ to increase the percentage of parasitized RBCs, as described (Levy and Ristic, 1980; Ueti et al., 2021). After 3-5 days of *in vitro* incubation, the culture media was removed by centrifugation at 2800 ×g for 3 min, cells were suspended in TRIzol^®^ reagent (Life technologies, Carlsbad, CA), and stored at -80 **°C** until used.

For obtaining kinetes, replete female ticks that fed on calves infected with *B. bigemina* during an ascending parasitemia were collected daily beginning at 6 days post-infection. Ticks were cleaned and rinsed in warm running water, distributed in 24-well plates, placed in containers with saturated KNO_3_ solution, and incubated at 26 **°C** at 92% relative humidity for 6 days to allow accumulation of *B. bigemina* kinetes in the tick’s hemolymph as previously described (Howell et al., 2007; Bohaliga et al., 2019). To identify ticks infected with *B. bigemina*, a distal leg segment was removed, a drop of exuding hemolymph was collected, put on a slide and Giemsa stained to examine for the presence of kinetes by light microscopy. *Babesia bigemina* kinetes were collected from ticks with >50 kinetes/hemolymph as previously described (Howell et al., 2007). The hemolymph samples were collected, pooled, concentrated by centrifugation at 4000 ×g for 2 min (Bohaliga et al., 2019), suspended in TRIzol reagent, and stored at -80 **°C** until used.

### RNA extraction and quality control

Parasite samples were suspended in TRIzol Reagent for total RNA extraction. Total RNA was extracted individually from paired blood-stage and kinete samples and treated with TURBO DNase (ThermoFisher Scientific) according to the manufacturer’s instructions to eliminate contaminating genomic DNA. RNA isolation was accomplished using the RNA Cleanup and Concentrator kit (RNA-5 and RNA-25) (Zymo Research, Irvine, CA) according to the manufacturer’s instructions. RNA concentration was measured by Nanodrop spectrophotometer (ThermoFisher Scientific, ND 1000) and tested for residual DNA by PCR targeting the CCp1 gene (Bastos et al., 2013). RNA quality was assessed by Nanodrop absorbances at 260/280 nm and 260/230 nm and Agilent Bioanalyzer Nano RNA chip (Agilent Technologies, Santa Clara, CA, USA), following the manufacturer’s recommendations. The RNA integrity number values for blood-stages were 9.5, 10 and 9.3 and kinetes were 9.7, 7.9 and 8.8. Purified RNA samples were stored at -80 **°C** until used.

### RNA-seq library preparation and sequencing

The RNA-seq libraries were constructed using the Illumina TruSeq mRNA stranded protocols (Illumina San Diego, CA, USA) following manufacturer’s recommendation. Briefly, from the total RNA, messenger RNA (mRNA) was enriched using poly-A selection, mRNA was chemically fragmented and denatured before transcription into first-stranded cDNA followed by double-stranded cDNA. Double-stranded cDNA was end-repaired and polyadenylated. Illumina dual index adapters were ligated to the cDNA samples followed by 9 cycles of PCR amplification. The libraries were quantified, normalized, and then multiplexed as previously described (Ueti et al., 2021). The multiplexed samples were sequenced on three lanes of a HiSeq 2500 (Illumina). Approximately 60 million total reads per sample were obtained. FASTQ files were generated with pipelines for the removal of adapter sequences from the 3’ ends of reads.

Differential expression analysis was performed at the Fred Hutchinson Cancer Institute (Seattle, WA, USA). The combined RNA-seq run resulted in six separate fastq files representing each library, containing sequenced paired reads and quality control data. The HiSeq Control software HCS 2.2.58 version and Real Time Analysis software RTA 1.18.64 version, supplied by Illumina Inc, were used. All raw and processed RNA sequencing data generated in this study have been submitted to the NCBI Gene Expression Omnibus (https://www.ncbi.nlm.nih.gov/geo/) under accession number GSE214115.

### Identification and analysis of differentially expressed genes

Differential gene expression analysis between kinetes and blood-stages was performed as follows. Low quality reads were filtered before alignment to the *B. bigemina* BOND reference genome (NCBIRefSeq GCF_000981445.1) using STAR v2.5.2a (2-pass mapping) (Dobin et al., 2013). Counts were generated from alignments for each gene using the Subread feature of Counts v1.6.0 (https://spark.apache.org/docs/1.6.0/programming-guide.html). Genes without at least 1 read per million mapped reads across all three samples within a group were removed, data was normalized using the Trimmed Mean of M-Values (TMM method) and analyzed for differential expression significance testing using the generalized linear model likelihood ratio test (GLM LRT) method in edgeR v3.20.9 (https://bioconductor.stastistik.tu-dortmund.de/packages/3.6/bioc/html/edgeR.html) (Robinson et al., 2010; McCarthy et al., 2012; Chen et al., 2016). The false discovery rate (FDR) method was employed to correct for multiple testing and genes were termed significantly differentially expressed if their log Fold Change (logFC) value was greater than or equal to 1 and the FDR set to 5%. The *p*-values were adjusted for multiple testing according to FDR correction (Benjamini and Hochberg, 1995).

### *In silico* analysis of genes in tick-borne pathogens

Orthologs of *B. bigemina* proteins were identified for *B. bovis*, *Babesia ovata* and *B. divergens* using the PiroplasmaDB resource (https://piroplasmadb.org/piro/app). Locus tags for *B. bigemina* genes were searched and the synteny genes were identified. Using the syntenic sequences track of the genome browser allowed the identification of orthologs among related Apicomplexan species.

Genes upregulated in *B. bigemina* kinetes was translated to proteins and used as queries to select potentially homologous sequences from *B. bovis* (T2Bo) [taxid:484906], *Theileria* spp. [taxid:5873] (that include *Theileria annulata*, *Theileria parva*, *Theileria orientalis* and *Theileria equi*), and *B. microti* [taxid:5868] based on the results of the protein BLAST platform (https://blast.ncbi.nlm.nih.gov/Blast.cgi) against the non-redundant protein sequences database. Sequences identified by BLASTp analyses were extracted using a custom script, and all results were manually inspected.

### Validation of RNA-seq data using RT-qPCR

Three hundred nanograms of the total RNA extracted from each preparation of parasite were treated with DNase I (Invitrogen, USA) to eliminate contaminating genomic DNA, and reverse transcribed (RT) using a SuperScript III kit. The resulting cDNA was used as a template in RT-qPCR with the SsoFast™ EvaGreen^®^ Supermix (BioRad, CA, USA). Gene expression was quantified on a CFX96™ Real-Time PCR Detection System (Bio-RAD). The amplification reaction was performed in a final volume of 20 µL using 0.8 µL of 10 µM of each primer set, 6.9 µL of nuclease-free water, 1.5 µL of a cDNA as template and 10 µL of SsoFast EvaGreen Supermix. The qPCR conditions consisted of an initial denaturation cycle at 95 °C for 3 min, followed by 40 cycles at 95 °C for 30 sec for denaturation, 60 °C for 30 sec for annealing, and 72 °C for 30 sec for extension. The specific primers (**Supplementary Table 1**) were designed using Primer3 software (Rozen and Skaletsky, 2000) and synthesized by Eurofins Genomics (Louisville, KY, USA). The specificity of qPCR was assessed by melting curve analysis. To confirm the nucleotide sequence of the specific DNA fragments for each designed primer, all amplicons were sequenced (data not shown). CFX Manager Software (Bio-Rad) was used to obtain quantification cycle (Cq) values (Bustin et al., 2009) and analyze expression data. The transcription level was calculated as a relative expression using 2^-ΔΔCq^ method (Livak and Schmittgen, 2001) to calculate the relative expression of the target gene after normalization to two reference genes, Mitogen-activated protein kinase (MAPK) and Proteosome A (ProtA) (**Supplementary Table 1**). These reference genes were selected from the RNA-seq data, as there was no variation in expression between the two groups evaluated (kinetes and blood-stages).

## Results and discussion

### RNA-seq analysis: Differential gene expression

**Figure 1:**
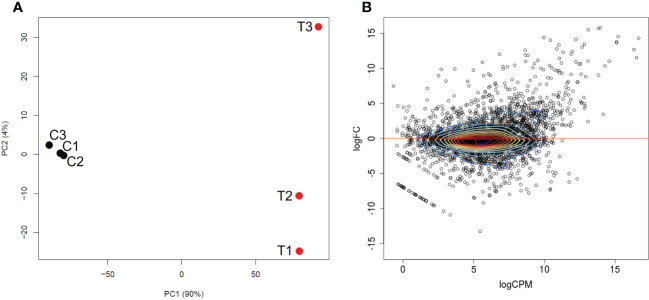
Differential transcriptional expression by *B. bigemina* blood-stages versus kinetes. **(A)** PCA plot between *B. bigemina* stages from blood (C1, C2, C3) and versus kinetes (T1, T2, T3). C indicates samples from calves and T indicates samples from ticks. **(B)** A MA plot shows the relationship of the log_2_ fold-change (FC) of *B. bigemina* blood-stages and kinetes versus the log_2_ normalized mean expression counts per million (CPM).

Approximately 120 million reads mapped to *B. bigemina* sequences were obtained from the analysis, of which 92 million corresponded to blood-stages, and 27 million to kinetes (**Supplementary Table 2**). Low rRNA rates were obtained (**Supplementary Table 2**), indicating the successful enrichment of mRNA from all blood and tick samples. Principal component analysis of the RNA-seq data resulted in separation of the biological replicates of the same condition into two distinct groups corresponding to blood-stages and kinetes (**Figure 1A**). The first principal component explained 90% of the variation between groups and the second principal component showed that blood-stage transcripts were more similar to each other as compared to kinete transcripts (**Figure 1A**). This pattern was observed in a previous *B. bovis* blood-stage and kinete RNA-seq study (Ueti et al., 2021) and may be associated with individual variability of samples due to the presence of tick hemocyte mRNA.

A total of 3,721 genes were identified in both *Babesia* stages (**Figure 1B**, **Table 1**). **Supplemental Data 1** provides a list of all differentially expressed genes (DEGs) found between *B. bigemina* kinetes and blood-stages. Among the DEGs, 906 genes were upregulated in kinetes, and 904 by blood-stages (**Table 1**). Interestingly, the magnitude of gene expression was greater in the kinete, reaching over ~56,000-fold increase as compared to the blood-stages which had only an ~9,600-fold increase. For comparison, the magnitude of expression in the *B. bovis* RNA-seq was >19,000-fold in kinetes and <300-fold increase in blood-stages (Ueti et al., 2021). **Table 1** shows the comparative gene expression in *B. bigemina* and *B. bovis*.

**Table 1:** Comparative transcription levels between *B. bigemina* and *B. bovis* kinetes and blood-stages.

	*Babesia bigemina*		*Babesia bovis*	
Fold change	Kinetes	Blood-stages	Kinetes	Blood-stages
>20,000	6	0	0	0
1,000 – 20,000	36	3	43	0
100 – 1,000	53	47	88	52
10 – 100	243	157	264	229
2 – 10	568	697	602	815
Total	906	904	997	1096
**Total genes**	3721		3887	

In *B. bigemina* kinetes, 42 genes had a fold change ranging from ~1,000 to 56,767, while in blood-stages, only 3 genes were upregulated in this range (**Table 1**). These kinete upregulated genes may be linked to the importance of these genes in biological functions related to invasion and survival of the parasite within the host tick. However, it can be speculated that upregulated genes may not be associated with protein expression as previously described (Lindner et al., 2019). A study in *Plasmodium* sporozoites analyzed highly abundant transcripts and correlated them with no or low amounts of detected protein, evidencing translational repression (Lindner et al., 2019). To understand more completely the biological importance that a given gene may have, studies at both the proteomic and functional levels must be also included. Furthermore, it is important to point out that gene products from less abundant transcripts may also be essential for parasite function.

In *B. bigemina*, highly expressed genes found in kinetes (over 1,000-fold increase) included kinete specific protein (KSP), rhomboid 4 (ROM4), membrane and RNA-binding protein, thrombospondin-related anonymous protein (TRAP), 6-cysteine (6-cys), inner membrane complex family protein, vesicle coat complex membrane and Der1-like family (Der-1) (**Table 2**). Orthologs of these genes were found in *B. bovis, B. ovata* and *B. divergens* (**Table 2**). Some highly expressed genes in *B. bigemina* kinetes were similar to *B. bovis* kinetes (**Table 2**), suggesting that these genes may play important roles during *Babesia* development within tick hemolymph including the invasion of the ovary and egg mass and may serve as potential candidate targets for the development of transmission blocking vaccines or drugs (Bohaliga et al., 2019). Gene expression by species is categorized in **Table 2**.

**Table 2:** Differential expression of genes highly expressed in *B. bigemina* kinetes. Orthologs of these genes in *B. bovis* (BBOV), *B. ovata* (BOVATA) and *B. divergens* (Bdiv) were found by synteny using PiroplasmaDB.

Gene ID	Annotation	Fold increase in *B. bigemina* kinetes	*B. bovis* ortholog	Identity to *B. bovis* (protein)	Fold increase in *B. bovis* kinetes	Other *Babesia* spp. ortholog	*B. bigemina* species-specific gene
BBBOND_0306920	Protein of unknown function with DNA repair domain	56767.20	none	–	–	BOVATA_003510	
BBBOND_0304920	Putative integral membrane protein	52812.63	BBOV_III003000	58%	9127.57		
BBBOND_0312490	Protein of unknown function	46963.41	none	–	–	BOVATA_001300	
BBBOND_0103540	6-Cys (D)	23586.46	BBOV_II006630	36%	878.82		
BBBOND_0208880	Protein of unknown function	21752.64	BBOV_IV005870	27%	7432.57	BOVATA_011290	
BBBOND_0206730	Kinete stage-specific protein	20159.79	BBOV_I002220	25%	19316.39	BOVATA_028050	
BBBOND_0108780	Protein of unknown function	17454.75	none	–	–	Bdiv_023800	
BBBOND_0102050	Protein of unknown function	16622.82	none	–	–	Bdiv_017050	
BBBOND_0104040	Rhomboid 4	14116.34	BBOV_II006100	66%	13286.23		
BBBOND_0103570	6-Cys (A)	12972.27	BBOV_II006600	42%	18031.07		
BBBOND_0311070	Protein of unknown function	11207.01	BBOV_III004080	33%	48.73	BOVATA_007380	
BBBOND_0402020	Protein of unknown function with large tegument domain	10343.05	none	–	–		
BBBOND_0308030	Putative membrane protein	8464.96	none	–	–	BOVATA_004660	
BBBOND_0311480	6-cys	7008.61	none	–	–		
BBBOND_0403750	Membrane protein	6655.79	BBOV_IV000290	56%	4272.74		
BBBOND_0300610	Protein of unknown function	6244.72	none	–	–	no other species have orthologs	
BBBOND_0212010	Protein of unknown function	5348.24	BBOV_II000160	45%	4.49		
BBBOND_0402310	Protein of unknown function with exodeoxyribonuclease domain	5018.25	none	–	–	BOVATA_036830	
BBBOND_0208480	RNA-binding protein	4171.06	none	–	–	Bdiv_027060c	
BBBOND_0106750	Protein of unknown function with RNA-binding domain	4164.59	BBOV_IV009220	67%	412.39		
BBBOND_0110360	Protein of unknown function	3850.04	BBOV_IV011940	26%	684.04		
BBBOND_0204020	Protein of unknown function	3684.81	BBOV_II003770	65%	292.45		
BBBOND_0202740	Thrombospondin-related anonymous protein	2963.64	BBOV_II002650	57%	1705.04		
BBBOND_0302080	6-cys	2745.36	none	–	–	BOVATA_045830	
BBBOND_0107490	Protein of unknown function	2273.91	none	–	–	no other species have orthologs	
BBBOND_0311160	Protein of unknown function	2101.10	none	–	–	BOVATA_007290	
BBBOND_0402880	Protein of unknown function	2076.11	none	–	–	no other species have orthologs	
BBBOND_0403740	Der1-like family	2070.65	BBOV_IV000300	61%	1690.43		
BBBOND_0405260	6-cys	1937.38	none	–	–	BOVATA_039640	
BBBOND_0204030	Protein of unknown function with methyltransferase domain family protein	1855.15	none	–	–	BOVATA_030310	
BBBOND_0208040	Inner membrane complex family protein	1731.23	BBOV_I003730	45%	685.47		
BBBOND_0405500	Vesicle coat complex membrane	1676.85	none	–	–	BOVATA_039420	
BBBOND_0306310	Protein of unknown function	1591.59	BBOV_III007330	55%	2624.29		
BBBOND_0209750	Protein of unknown function	1590.83	BBOV_IV005140	42%	17381.30		
BBBOND_0102540	Protein of unknown function	1392.16	none	–	–	Bdiv_014440c	
BBBOND_0401710	Protein of unknown function	1354.80	BBOV_IV006480	28%	7388.35		
BBBOND_0310840	Protein of unknown function	1311.59	none	–	–	BOVATA_007230	
BBBOND_0313010	Protein of unknown function with WD domain	1223.30	none	–	–	Bdiv_032120c	
BBBOND_0103640	membrane protein	1204.07	BBOV_II006530	50%	209.18		
BBBOND_0104060	6-cys	1103.92	none	–	–	BOVATA_022080	
BBBOND_0100160	6-cys	1100.28	none	–	–	no other species have orthologs	
BBBOND_0101590	6-cys	1023.04	none	–	–	no other species have orthologs	

Highlighted in yellow are the genes with expression that was similar in kinetes of *B. bigemina* and *B. bovis.* In green, gene expression that was higher in *B. bigemina* as compared to *B. bovis.* In blue, gene expression that was higher in *B. bovis* as compared to *B. bigemina* and in white, *B. bigemina* genes without orthologs in *B. bovis.*

In *B. bigemina* blood-stages, 43 genes with increased expression (>120-fold change) were identified (**Table 3**). In addition to many proteins of unknown function, two ribosome-binding proteins and one probable fructokinase were found in *B. bigemina* blood-stages (**Table 3**). The functions of the gene products of most of the upregulated genes have not yet been characterized and may have biological importance related to invasion, gliding motility, and egress (González et al., 2019). Expression of two *B. bigemina* blood-stage genes exceeded that of *B. bovis* orthologs (**Table 3**).

**Table 3:** Differential expression of genes with over 120-fold increase in B. bigemina blood-stages. Orthologs of these genes were found in B. bovis (BBOV) and B. ovata (BOVATA) by synteny using PiroplasmaDB.

Gene ID	Annotation	Fold increase in *B. bigemina* blood-stages	*B. bovis* ortholog	Identity to *B. bovis* (protein)	Fold increase in *B. bovis* blood-stages	Other *Babesia* spp. ortholog	*B. bigemina* species-specific gene
BBBOND_0205880	Protein of unknown function with homoserine dehydrogenase family domain	9686.43	none	–	–		✓
BBBOND_0001870	Protein of unknown function with reverse transcriptase like family domain	1931.22	cannot determine				
BBBOND_0400070	Protein of unknown function	1314.89	none	–	–	BOVATA_039000	
BBBOND_0100220	Protein of unknown function with Collagen superfamily and Trichohyalin-plectin-homology domains	738.29	none	–	–		✓
BBBOND_0111020	Protein of unknown function	673.15	cannot determine				
BBBOND_0101920	Protein of unknown function	591.82	none	–	–	BOVATA_024760	
BBBOND_0313390	Protein of unknown function	488.21	none	–	–	BOVATA_000410	
BBBOND_0300580	Protein of unknown function	485.22	none	–	–		✓
BBBOND_0404420	Protein of unknown function	478.10	none	–	–		✓
BBBOND_0006320	Protein of unknown function	430.22	cannot determine				
BBBOND_0000700	Protein of unknown function with Smc Chromosome segregation ATPase domain	410.33	cannot determine				
BBBOND_0312120	Protein of unknown function with M protein C-terminal and large tegument protein UL36 domains	372.82	none	–	–		✓
BBBOND_0405420	Protein of unknown function	361.35	none	–	–	BOVATA_039490	
BBBOND_0109490	Protein of unknown function	347.82	none	–	–		✓
BBBOND_0210450	Protein of unknown function	345.29	none	–	–	BOVATA_012660	
BBBOND_0005400	Protein of unknown function with reticulocyte binding/rhoptry protein domain	335.59	cannot determine				
BBBOND_0207850	Protein of unknown function	290.86	none	–	–	BOVATA_026980	
BBBOND_0201910	Protein of unknown function	268.80	none	–	–	BOVATA_032350	
BBBOND_0307370	Protein of unknown function	268.74	none	–	–	BOVATA_004020	
BBBOND_0208770	Protein of unknown function	261.63	none	–	–	BOVATA_011160	
BBBOND_0005370	Protein of unknown function with endonuclease subunit and DNA repair exonuclease SbcCD ATPase subunit domains	261.49	cannot determine				
BBBOND_0307380	Protein of unknown function	254.89	none	–	–		
BBBOND_0305790	Protein of unknown function	251.91	none	–	–	BOVATA_002330	
BBBOND_0000630	Protein of unknown function with reticulocyte binding/rhoptry protein domain	241.53	cannot determine				
BBBOND_0204010	Protein of unknown function	220.63	BBOV_II003760	35.79	33.84		
BBBOND_0005840	Protein of unknown function with Apolipophorin-III like domain	211.45	cannot determine				
BBBOND_0202980	Protein of unknown function with ribonuclease E domain	195.31	none	–	–	BOVATA_033390	
BBBOND_0402550	Protein of unknown function	192.57	none	–	–	BOVATA_036600	
BBBOND_0304560	Protein of unknown function with reticulocyte binding/rhoptry protein domain	182.60	none	–	–	BOVATA_008880	
BBBOND_0400010	Protein of unknown function with chromosome segregation protein SMC and reticulocyte binding/rhoptry protein domains	178.94	none	–	–	BOVATA_046230	
BBBOND_0313950	Protein of unknown function with Smc Chromosome segregation ATPase and DNA repair exonuclease SbcCD ATPase subunit domains	177.52	none	–	–		
BBBOND_0306390	Protein of unknown function	177.33	none	–	–	BOVATA_003120	
BBBOND_0300430	Ribosome-binding protein 1	174.63	none	–	–		✓
BBBOND_0100430	Probable fructokinase	161.13	BBOV_IV003810	34.72	36.64		
BBBOND_0208830	Protein of unknown function	151.61	none	–	–	BOVATA_011240	
BBBOND_0101800	Protein of unknown function with Peptidase C1A subfamily and Cathepsin propeptide inhibitor domain (I29) domains	149.56	none	–	–	BOVATA_024640	
BBBOND_0306680	Protein of unknown function	141.63	none	–	–	BOVATA_002840	
BBBOND_0313380	Ribosome-binding protein 1	136.12	none	–	–	BOVATA_000440	
BBBOND_0005690	Protein of unknown function with Smc Chromosome segregation ATPase and DNA double-strand break repair ATPase Rad50 domains	128.63	cannot determine				
BBBOND_0004810	Protein of unknown function with Reverse transcriptase like family domain	128.41	cannot determine				
BBBOND_0006030	Protein of unknown function with Smc Chromosome segregation ATPase	127.32	cannot determine				
BBBOND_0110820	Protein of unknown function	122.12	none	–	–	BOVATA_015600	
BBBOND_0005760	Protein of unknown function	120.60	cannot determine				

Highlighted in green, gene expression was higher in B. bigemina compared to B. bovis and in white, B. bigemina genes with no orthologs in B. bovis.

It is interesting to note that of the 42 highly expressed genes in *B. bigemina* kinetes, 20 had orthologs in *B. bovis*. However, this was not the case for blood-stages, as only two of the 43 genes upregulated by >120-fold had orthologs in *B. bovis* (**Tables 2** and **3**). In the RNA-seq of *B. bovis*, differentially expressed kinete genes with levels >1,000-fold change were KSP, ROM family proteins, membrane proteins, TRAP, 6-cys, protein kinases, Der-1, GCC2/GCC3 protein, and AP2 families (Ueti et al., 2020; Ueti et al., 2021). Importantly, of the genes that exceeded >1,000-fold change, 34 genes were unique for *Babesia* when compared to the bovine genome by BLASTp (data not shown).

The list of *B. bovis* genes expressed by blood-stages (>120-fold increase) is quite distinct from those genes expressed by *B. bigemina* during the same life cycle phase in the mammalian host. In *B. bovis* blood-stages, small open reading frame (SmORF), variant erythrocyte surface antigen (VES), merozoite surface antigen (MSA), spherical body protein (SBP), membrane proteins, ROM family proteins, rhoptry-associated protein 1 (RAP-1) family protein and others were upregulated (Ueti et al., 2020; Ueti et al., 2021). *B. bovis* infected RBC (iRBC), but not *B. bigemina* iRBC, can adhere to vascular endothelial cells and accumulate in the host microvasculature, a process that is apparently mediated by VES proteins. *Babesia bovis* changes the protein and lipid composition of the iRBC membrane, modifying its architecture resulting in increased cellular adhesiveness and high antigenic variability (Suarez et al., 2019). The VES gene family is also responsible for this antigenic variation, and this phenomenon, combined with epithelial cell cytoadhesion by iRBCs, allows *B. bovis* to evade the vertebrate host immune response. The *B. bovis* genome contains 133 members of VES (Allred et al., 2000; Eichenberger et al., 2017; Ueti et al., 2021). However, the role of VES proteins in *B. bigemina* is unknown and understanding its mechanisms may be a key to potential vaccine candidates (Suarez et al., 2019). (Jackson et al., 2014) described some VES genes in *B. bigemina* BOND, JG29, Puerto Rico, and BbiS3P strains, however, in our RNA-seq experiments no differential expression of VES genes was found in either kinetes or blood-stages. In addition, the SmORF genes may assist in capillary sequestration (Suarez et al., 2019). However, SmORF genes are present only in *B. bovis*, and not in *B. bigemina* (Suarez et al., 2019). Altogether, these differences in gene expression may contribute to increased virulence and pathogenicity of *B. bovis* when compared to *B. bigemina*.

Synteny analysis using the Piroplasma genome database showed that nine genes classified as protein of unknown function, three 6-cys and one ribosome-binding protein 1 had no orthologs in *B. ovata* and *B. divergens*, suggesting that they are *B. bigemina*-specific genes (**Tables 2** and **3**).

### *Babesia bigemina* differentially expressed genes

#### HAP2/GCS1 domain protein

The fusion of male and female gametes to generate a zygote is an important step in the life cycle of many organisms and has been considered a target for transmission blocking vaccines. The gene HAP2/GCS1 (hapless 2/generative cell specific 1) was identified in plants, *Plasmodium*, and other organisms (Johnson et al., 2004; Mori et al., 2006; Hirai et al., 2008) as important keys in gamete fertilization. In bovine *Babesia*, HAP2 was found to be essential for the development of sexual forms in *hap2* KO parasites and expressed on the surface of *B. bovis* gametes (Hussein et al., 2017). Interestingly, anti-HAP2 antibodies blocked *B. bigemina* zygote formation (Camacho-Nuez et al., 2017). The expression of HAP2-GCS1 was increased 20-fold in *B. bigemina* kinetes (BBBOND_0307410). This gene was also increased in *B. bovis* kinetes (BBOV_III006770, 258-fold) (Ueti et al., 2021), but 13 times more than *B. bigemina.* HAP2 is a gamete fusion protein and may be is involved in attachment of kinetes to ovary epithelial cells (Ueti et al., 2021). However, further investigation is needed to test if HAP2 protein is expressed on the surface of *Babesia* kinetes.

#### CCp family

The CCp gene family is related to the development of sexual stages in Apicomplexa organisms, including *Plasmodium*, *Theileria*, and *Babesia* (Pradel et al., 2004; Becker et al., 2010; Bastos et al., 2013). In the *B. bigemina* RNA-seq, three members of the CCp family, named CCp1, CCp2 and CCp3 (BBBOND_0307800, BBBOND_0203950 and, BBBOND_0312400, respectively), containing LCCL (*Limulus* coagulation factor C) motifs, were upregulated in blood-stages, which ranged from 1.8 to 8.9-fold. The same pattern was observed in *B. bovis* (BBOV_III006360, BBOV_II003700, and BBOV_III008930) with blood-stage expression varying from 2.2 to 8.8-fold (Ueti et al., 2021). In a previous study, the gene expression and proteins of CCp1-3 was proven in Puerto Rico strain *B. bigemina* induced sexual stages (Bastos et al., 2013). A recent study showed that in addition to the described CCp1-3, two novel non-canonical members of the CCp gene family, named CCp5 and FNPA, were also found in *B. bovis*. Both genes are highly conserved among geographically distinct *B. bovis* strains (Ozubek et al., 2022). *Babesia bovis* FNPA protein has a predicted transmembrane domain, and this fact characterizes it as a possible target of a transmission-blocking vaccine against *B. bovis* (Ozubek et al., 2022). In the *B. bigemina* and *B. bovis* blood-stages RNA-seqs, upregulation of CCp5 (BBBOND_0306080, 2.8-fold and BBOV_III007200, 2-fold) and FNPA (BBBOND_0206110, 3.4-fold and BBOV_I002720, 2.8-fold increase) (Ueti et al., 2021) were found.

#### GCC2/GCC3 domain containing protein family

The GCC2/GCC3 domain containing proteins belong to the cysteine repeat modular proteins (CRMPs) family, which is an important specific protein of apicomplexan parasites. In *Plasmodium*, it was postulated that all four CRMP are required for host cell interaction. Analysis of *P. berghei* sporozoites lacking PCRMP1 – 4, showed they were incapable of invading the mosquito salivary gland. In addition, mutant sporozoites lacking PCRMP3 or 4 did not proliferate inside hepatocytes (Douradinha et al., 2011). In *Plasmodium* GCC2/GCC3 are differentially expressed in pairs during the parasite life cycle. This was also found to be true for *B. bovis* where different GCC2/GCC3 pairs were upregulated based on life stage. In *B. bovis* kinetes, BBOV_III011730, 761-fold and BBOV_IV006250, 1,082-fold, were upregulated while BBOV_III011740, 5.6-fold and BBOV_IV006260, 14-fold were upregulated in blood-stages (Ueti et al., 2021). In contrast, in the *B. bigemina* RNA-seq, three kinete genes were up-regulated (BBBOND_0208310 and BBBOND_0208320 (both 98-fold) and BBBOND_0404220 (137-fold)). In *B. bovis*, GCC2/GCC3 genes are arranged in tandem, however, the BOND annotation indicated an intervening gene between BBBOND_0208300 and BBBOND_0208320. *In silico* and sequencing analysis of RT-PCR amplicons using forward and reverse primers spanning BBBOND_0208310 and BBBOND_0208320, respectively, suggested that there was not an intervening gene, and that the gene model may need to be updated (**Supplementary Figure 1**). Expression of GCC2/GCC3 proteins in *B. bigemina* blood-stages did not follow the paired upregulation of *B. bovis* as only a single protein, BBBOND_0404210 (1.8-fold), was upregulated, and another, BBBOND_0208300, had no difference in expression.

#### The AP2 family

The Apetala 2 (AP2) family are nuclear transcription factors and contain conserved structural motifs that are directly involved in DNA binding (Alzan et al., 2016). AP2 encompasses the main transcription factors specific to Apicomplexa (Balaji et al., 2005). These genes are associated with transcriptional control of other genes involved in parasite development (Suarez et al., 2019). Several described *B. bigemina* genes encoding proteins containing AP2 domains (Alzan et al., 2016) and found in *B. bovis* (Ueti et al., 2021) were differentially expressed in *B. bigemina* blood-stage and kinetes. **Table 4** identifies the *B. bigemina* AP2 genes that had orthologs in *B. bovis*, although their architectures may show considerable variability (28 - 74% identity). In *B. bigemina*, there are a total of 23 AP2 genes that were identified. Eleven were upregulated in *B. bigemina* kinetes and five in the blood-stages, while seven were not different between groups (**Table 4**), suggesting that they could be differentially expressed by forms at other points of the biological cycle, such as gametes or sporozoites. The *B. bigemina* AP2 gene-family expression profile suggests the highest expression level for BBBOND_0104820 in blood-stage parasites (**Table 5**). Its ortholog BBOV_II005480 was also the highest expressed AP2 in *B. bovis* blood-stages (Ueti et al., 2021). However, in kinetes, the correlation between *B. bigemina* and *B. bovis* was not maintained. Interestingly, *B. bigemina* BBBOND_0312340 and BBBOND_0403500 AP2 genes were upregulated in blood-stages but their orthologs in *B. bovis* (BBOV_III008870 and BBOV_IV000500) were upregulated in kinetes. Further investigation of the functions of the AP2 genes in these two *Babesia* are necessary to understand why the pattern of expression of these two AP2 genes is distinct between *B. bigemina* and *B. bovis*. This differential expression may be linked to differences in virulence or the needs of the parasite at different stages in its life cycle (in different hosts). *Babesia bigemina* kinetes had a higher magnitude of gene expression when compared to *B. bovis* (**Table 1**) and perhaps differences in AP2 genes contribute to the transcriptional control of other genes required by kinetes for survival/development/interaction in the invertebrate host.

**Table 4:** AP2 gene family expressed in kinetes and blood-stages in *B. bigemina* and their respective orthologs in *B. bovis*.

Gene ID	Upregulated in *B. bigemina*	*B. bovis* ortholog	Identity to *B. bovis*
BBBOND_0301220	Kinetes	BBOV_I000100	55%
BBBOND_0206620	Kinetes	BBOV_I002320	56%
BBBOND_0207810	no significance	BBOV_I003560	47%
BBBOND_0402120	Kinetes	BBOV_I004280	37%
BBBOND_0405860	Kinetes	BBOV_I004850	74%
BBBOND_0201480	no significance	BBOV_II001610	55%
BBBOND_0203280	Blood-stages	BBOV_II003230	45%
BBBOND_0204230	Kinetes	BBOV_II004230	58%
BBBOND_0104820	Blood-stages	BBOV_II005480	59%
BBBOND_0103120	Blood-stages	BBOV_II007120	40%
BBBOND_0301930	no significance	BBOV_III000570	63%
BBBOND_0311370	no significance	BBOV_III003770	44%
BBBOND_0311080	Kinetes	BBOV_III004090	73%
BBBOND_0310100	Kinetes	BBOV_III004740	50%
BBBOND_0307130	Kinetes	BBOV_III007040	55%
BBBOND_0306760	no significance	BBOV_III007880	58%
BBBOND_0312340	Blood-stages	BBOV_III008870	68%
BBBOND_0313300	no significance	BBOV_III009600	42%
BBBOND_0403500	Blood-stages	BBOV_IV000500	28%
BBBOND_0404460	Kinetes	BBOV_IV002360	64%
BBBOND_0109970	Kinetes	BBOV_IV011690	33%
BBBOND_0110120	Kinetes	BBOV_IV011830	40%
BBBOND_0301490	no significance	BBOV_III000230	59%

**Table 5:** AP2 gene family expressed in blood-stages and in kinetes in *B. bigemina*.

Gene ID	Blood-stages CPM	Kinetes CPM
BBBOND_0104820	3746.16 *	631.84
BBBOND_0312340	823.09 *	144.79
BBBOND_0204230	590.83	1551.25 *
BBBOND_0201480	464.44	685.82
BBBOND_0403500	431.89 *	194.77
BBBOND_0103120	403.97 *	105.80
BBBOND_0306760	243.48	239.94
BBBOND_0110120	205.20	571.44 *
BBBOND_0301490	141.50	201.06
BBBOND_0313300	124.43	202.45
BBBOND_0203280	88.94 *	43.59
BBBOND_0311370	82.40	120.07
BBBOND_0207810	71.77	97.39
BBBOND_0307130	64.36	602.07 *
BBBOND_0206620	45.72	668.23 *
BBBOND_0311080	17.59	528.57 *
BBBOND_0405860	34.27	15543.77 *
BBBOND_0402120	13.44	7577.71 *
BBBOND_0301220	5.08	4158.92 *
BBBOND_0109970	5.08	4158.92 *
BBBOND_0404460	3.73	150.20 *
BBBOND_0301930	3.61	4.61
BBBOND_0310100	3.40	31.07 *

CPM is counts per million and * indicates statistical significant differences (p<0.05).

#### 6-cys gene family

The 6-cys gene family was originally identified in *Plasmodium* and plays important roles in gamete stages and oocyte formation. Members of the 6-cys family are considered strong candidates for vaccine development (van Dijk et al., 2010; Alzan et al., 2017). These proteins are defined by domains that contain 6 positionally conserved cysteines, but arrangements of 4, 5 or 7 cysteine residues can also be found in *Babesia* (Alzan et al., 2016). In addition, most 6-cys proteins have signal peptides, suggesting these proteins are likely to be expressed on the surface of the parasite, or secreted (Alzan et al., 2016). In *B. bovis*, this family is composed of ten genes (A-J) that are related to the development of gametes in the *R. microplus* midgut (Alzan et al., 2016). *Babesia bovis* RNA-seq revealed that all ten 6-cys genes were upregulated in kinetes (Ueti et al., 2021). In fact, four members of 6-cys (C, A, B and E) (BBOV_II006620, BBOV_II006600, BBOV_II006610 and BBOV_II006640) were highly expressed in kinetes (19,316, 18,031, 14,454 and 1,731-fold increase respectively) (Ueti et al., 2021). In *B. bigemina*, some 6-Cys family members unique to *B. bigemina* or shared with a *B. ovata* ortholog were highly upregulated in kinetes (**Table 2**), suggesting that they may play a role during tick infection. The 6-Cys family is comprised of 11 members with ten upregulated in kinetes and a single member upregulated in blood-stages (**Table 6**). In *Plasmodium vivax*, three 6-Cys family proteins were expressed in the blood-stage and might be involved in merozoite invasion activity (Wang et al., 2014).

**Table 6:** Upregulated 6-Cys gene family in *B. bigemina* kinetes and blood-stages.

Gene ID	Stage	Annotation	Fold increase
BBBOND_0103540	Kinetes	6-CysD	23,586.46
BBBOND_0103570	Kinetes	6-CysA	12,972.27
BBBOND_0311480	Kinetes	6-Cys	7,008.61
BBBOND_0302080	Kinetes	6-Cys	2,745.36
BBBOND_0405260	Kinetes	6-Cys	1,937.38
BBBOND_0104060	Kinetes	6-Cys	1,103.92
BBBOND_0100160	Kinetes	6-Cys	1,100.28
BBBOND_0101590	Kinetes	6-Cys	1,023.04
BBBOND_0103530	Kinetes	6-CysE	524
BBBOND_0400600	Kinetes	6-CysJ	6
BBBOND_0400720	Blood-stages	6-CysI	1.7

#### ABC transporter

The ATP binding cassette (ABC) transporter genes are present in all prokaryotes and eukaryotes, including Apicomplexan parasites. This family of genes codes for different proteins which translocate amino acids, ions, peptides, proteins, cholesterol, metabolites, and toxins across extra- and intracellular membranes and contribute to drug resistance through decreased drug uptake into the cell or efflux (which expel toxins and drugs out of the cell) (El-Awady et al., 2016). Two putative ABC transporters were upregulated in *B. bigemina* kinetes (BBBOND_0403540 and BBBOND_0207340, both over 11-fold), and five members were upregulated in the blood-stage (BBBOND_0309830, BBBOND_0105520, BBBOND_0100850, BBBOND_0313150 and BBBOND_0300960) by 1.65 to 3-fold. In a *B. bovis* RNA-seq, ABC transporters were also upregulated in kinetes (BBOV_III006450, BBOV_III011170 and BBOV_I003180) and in blood-stages (BBOV_IV008140, BBOV_III005570, BBOV_I000600) (Ueti et al., 2021). The expression varied between 3.5 to 85.8-fold change. ABC transporter proteins are immunogenic and have been used as antigens in the formulation of vaccines against bacteria (Riquelme-Neira et al., 2013; Murphy et al., 2016). In protozoan parasites, like *P. falciparum* and *Leishmania* sp., the ABC transporters are targets of antiprotozoal drugs (Klokouzas et al., 2003; El-Awady et al., 2016).

#### Iron transporter

Iron receptors and transporters are associated with the pathogen’s virulence and immunogenicity and are usually located on the cell surface, making them good targets for drugs or vaccines (Glanfield et al., 2007). Iron-sulfur clusters (Fe-S) are small inorganic metal cofactors, and proteins that contain Fe-S clusters have functions that include electron transport, regulation of gene expression, substrate binding and activation, radical generation, and DNA repair (Wachnowsky et al., 2018). An important antiparasitic drug, artemisinin, has iron-dependent activity, and the artemisinin drug family is a potent first-line antimalarial drugs (Sibmooh et al., 2001; Olliaro and Taylor, 2004). Artesunate, a derivative of artemisin, effectively inhibited growth of cultures of *B. bovis* and *B. gibsoni* (Goo et al., 2013). The role of iron uptake and metabolism in *Babesia* should be better studied for new potential therapeutic targets. *Babesia bigemina* kinetes express an Fe-S cluster biosynthesis domain gene (BBBOND_0307200, 57-fold increase), Fe-S cluster assembly protein NFU-like (BBBOND_0211120, 2-fold) and CDGSH Fe-S domain (BBBOND_0202830, 2-fold). In a *B. bovis* RNA-seq, Fe-S cluster assembly scaffold family protein (BBOV_III007290, 2-fold and BBOV_IV004050, 3-fold) were found in blood-stages and kinetes (Ueti et al., 2021).

### Virulence genes

Sixteen virulence genes described and analyzed in the virulome of Mexican *B. bigemina* strain (Sachman-Ruiz et al., 2021) were found to be upregulated in kinetes and blood-stages (**Table 7**) in this RNA-seq. Most virulence genes are protein kinases (PKA), which is a family of enzymes that modifies other proteins by adding phosphates. The phosphorylation by PK has been reported to regulate important cellular processes such as transcription, translation, protein synthesis, cell cycle, and apoptosis in Apicomplexa parasites (Kato et al., 2012). Use of staurosporine, an inhibitor of serine/threonine protein kinase, causes the inhibition of host cell invasion or growth of several Apicomplexa species (*Plasmodium*, *Trypanosoma*, and *Leishmania*) (Bork et al., 2006). Furthermore, it led to the complete clearing of *B. bovis* parasitemia and caused a significant increase in the percentage of extracellular merozoites, probably due to inhibition of erythrocyte invasion by the parasite (Bork et al., 2006).

**Table 7:** Virulence genes in *B. bigemina* kinetes and blood-stages.

Gene ID	Stage	Annotation	Fold increase
BBBOND_0402050	Kinetes	cAMP-dependent protein kinase (PKA)	215.6
BBBOND_0211720	Kinetes	Transcription factor TFIIB subunit	58
BBBOND_0110960	Kinetes	Calcium-dependent protein kinase 4 (CDPK4)	31
BBBOND_0402030	Kinetes	Glycerol kinase (GK)	20
BBBOND_040253	Kinetes	Phosphatidylinositol-4-phosphate 5-kinase	13.5
BBBOND_0105020	Kinetes	Glycogen synthase kinase-3 alpha	11.3
BBBOND_0313410	Kinetes	Protein kinase domain containing protein	5.7
BBBOND_0305090	Kinetes	Casein kinase 1 (CK1)	4.2
BBBOND_0103000	Kinetes	Calmodulin-domain protein kinase 2	3.7
BBBOND_0102770	Kinetes	Transcription initiation factor TFIIB	2
BBBOND_0100430	Blood-stages	Probable fructokinase	161
BBBOND_0402770	Blood-stages	Protein Serine/Threonine kinase 1	4.8
BBBOND_0102860	Blood-stages	Serine/Threonine protein kinase	2.3
BBBOND_0309300	Blood-stages	Diphosphate kinase family	2
BBBOND_0205740	Blood-stages	Phosphatidylinositol 3-and 4-kinase	2
BBBOND_0110510	Blood-stages	MAPKK-related Serine/Threonine protein kinase	2
BBBOND_0406010	no significance	cGMP dependent protein kinase	–
BBBOND_0401620	no significance	Cyclin 4	–
BBBOND_0209200	no significance	Mitogen-activated protein kinase	–
BBBOND_0107050	no significance	Phosphatidylinositol 4 kinase	–
BBBOND_0101090	no significance	Phosphatidylinositol-4-phosphate 5-kinase family protein	–
BBBOND_0400240	no significance	Serine/Threonine protein kinase	–

In humans, the transcription factor TFIIB is phosphorylated *in vivo* at serine 65 and this phosphorylation is important for the transcription process (Wang et al., 2010). Unfortunately, no study reports the function of TFIIB in Apicomplexa parasites. However, in *B. bigemina*, TFIIB was upregulated in kinetes (**Table 7**) and in *B. bovis* (BBOV_II007500, 2.2-fold) (Ueti et al., 2021).

In *P. falciparum* gametocytes, whole genome microarray analysis identified glycerol kinase (GK) as the second most highly upregulated gene, and its function is linked to the phosphorylation of host-glycerol (Schnick et al., 2009). GK deletion from *P. falciparum* had no effect on asexual parasite growth, gametocyte development or exflagellation, suggesting that these life cycle stages do not utilize host-derived glycerol as a carbon source (Schnick et al., 2009). GK was upregulated in both *B. bigemina* (**Table 7**) and *B. bovis* kinetes (BBOV_I004170, 11-fold) (Ueti et al., 2021).

The virulence factor casein kinase I members are serine/threonine protein kinases, highly conserved in eukaryotes (protozoan to humans) and involved in various cellular processes such invasion, protein trafficking, transcription, cell cycle regulation, apoptosis, phosphorylation of upstream kinases, protein-protein interactions, and others (Rachidi et al., 2021). Some studies in *Plasmodium*, *Toxoplasma* and *Leishmania* evaluated the role of this protein and suggest CK1 was an important vaccine candidate, but to date, drug screening has only been studied in *Leishmania* (Rachidi et al., 2021). In *Leishmania*, CK1 has functions on host immune system, with phosphorylation in human C3a (complement system) and inducing immune evasion (Lamotte et al., 2017). In addition, CK1 inhibitors have been shown to block the growth of extracellular *Leishmania* in *in vitro* culture (Allocco et al., 2006). In *Babesia*, CK1 was upregulated in kinetes of *B. bigemina* (**Table 7**) and *B. bovis* (BBOV_III002900, 21.3-fold). Further studies should assess whether the protein encoded by this gene expressed in *Babesia* kinetes interacts with the tick’s innate immune system, leading to avoidance of the complement-like system and, thus, permitting success in infecting tick tissues.

Additionally, another six virulence genes cited in (Sachman-Ruiz et al., 2021) were identified, however, there was no difference between groups (**Table 7**). These virulence genes may be expressed in other phases of the biological cycle of *B. bigemina* and consequently, not evaluated in this dataset.

### *Babesia bigemina* genes related to host cell invasion and egress molecular processes

The mechanism of cell invasion of the *Babesia*-related apicomplexan parasites, including *Toxoplasma* and *Plasmodium*, involves initial pathogen attachment to molecules on the cell membrane of the target cell, followed by reorientation, leaving its apical pole facing the cell surface before the parasite actively penetrates the host cell. This active penetration mechanism utilizes an actin/myosin-based motor complex. Some specific structural characteristics of Apicomplexan parasites are related to their motility/migration and invasion/egress of host cells (Suarez et al., 2019). The parasites have an outer plasmatic membrane (PM) and an inner plasmatic complex (IMC), and the space between the two membranes forms the molecular complex (glideosome) that allows the parasite to move. Another important structure involved in the invading process of Apicomplexan parasites is an apical complex that includes several organelles such as rhoptries, micronemes and conoids. However, cell invasion by *Babesia* parasites does not involve conoids (Suarez et al., 2019).

### Gliding motility

*Babesia bigemina* kinete and blood-stage RNA-seq revealed 39 genes putatively related to the components of the molecular invasion/egress processes that were upregulated between 1.6- to 14,116.3-fold increase in kinetes and 1.7- to 56.7-fold change in blood-stages (**Table 8**). Of the components that make up the glideosome, actin, myosin A and gliding-associated protein 45 (GAP45) were upregulated in *B. bigemina* kinetes (**Table 8**). A previous *B. bovis* RNA-seq also found actin (BBOV_IV009790, and BBOV_I000300, with 16.6- and 12.6-fold increase in kinetes and BBOV_I004010, BBOV_IV003760, BBOV_IV005480, BBOV_III010900, with 2.2- to 3.7-fold increase in blood-stage), myosin A (BBOV_I003490, 9.8-fold) and GAP45 (BBOV_II005470, 2.3-fold increase) were increased (Ueti et al., 2021). *Babesia bigemina* actin, myosin A, and GAP45 have high identity with their *B. bovis* orthologs (63 to 98%) (**Table 8**). *Babesia bigemina* myosin B and myosin light chain (MLC) genes were found to be upregulated in blood-stages (**Table 8**), suggesting that the movement in *B. bigemina* could be governed by alternative myosin motors, similar to *B. divergens* and *P. falciparum* (Hernández et al., 2017; González et al., 2019). In the *B. bovis* RNA-seq, myosin B expression (BBOV_IV012030) was not significantly different between stages (Ueti et al., 2021).

**Table 8:** Genes related with invasion and egress processes in *B. bigemina* kinetes and blood-stages.

Gene ID	Up regulated	Annotation	Fold increase in *B. bigemina*	Gene ID (*B. bovis*)	Identity to *B. bovis* (protein)
BBBOND_0300760	Kinetes	Actin	10.94	BBOV_I000300	99%
BBBOND_0100390	Kinetes	Actin-related protein	1.55	BBOV_IV003760	58%
BBBOND_0209240	Blood-stages	Actin-related	1.75	BBOV_IV005480	59%
BBBOND_0207730	Kinetes	Myosin A	16.69	BBOV_I003490	82%
BBBOND_0306480	Blood-stages	Myosin light chain kinase	1.95	BBOV_III007480	55%
BBBOND_0110450	Blood-stages	Myosin B	23.23	BBOV_IV012030	68%
BBBOND_0104830	Kinetes	Gliding-associated protein 45	8.24	BBOV_II005470	63%
BBBOND_0102640	Kinetes	Calcium-dependent protein kinase (CDPK)	12.09	BBOV_II007640	54%
BBBOND_0110960	Kinetes	Calcium-dependent protein kinase 4 (CDPK4)	31.10	BBOV_IV003210	85%
BBBOND_0202740	Kinetes	Thrombospondin-related anonymous protein (TRAP1)	2,963.64	BBOV_II002650	61%
BBBOND_0202800	Blood-stages	Thrombospondin-related anonymous protein (TRAP)	13.49	BBOV_II002890 (TRAP2) BBOV_II002870 (TRAP4)	41% 29%
BBBOND_0202760	Blood-stages	Thrombospondin-related anonymous protein (TRAP3)	18.60	BBOV_II002630	38%
BBBOND_0301100	Kinetes	Serine/threonine protein phosphatase (PP2A)	34.33	BBOV_I000740	87%
BBBOND_0403910	Blood-stages	Serine/threonine protein phosphatase 5	2.33	BBOV_IV000160	78%
BBBOND_0201500	Kinetes	PPKL/Serine/threonine phosphatase	3.16	BBOV_II001630	81%
BBBOND_0104040	Kinetes	Rhomboid family protein 4 (ROM4.1)	14,116.33	BBOV_II006100	67%
BBBOND_0209020	Kinetes	Rhomboid family protein (ROM8)	2	BBOV_IV005790	60%
BBBOND_0301890	Blood-stages	Rhomboid family protein (ROM7)	1.65	BBOV_III000530	50%
BBBOND_0104210	Blood-stages	Rhomboid family protein (ROM4)	2.4	BBOV_II005940	41%
BBBOND_0310300	Kinetes	Rhomboid-related	71.73	no *B. bovi*s ortholog	N/A
BBBOND_0208040	Kinetes	Inner membrane complex family protein	1,731.23	BBOV_I003730	46%
BBBOND_0201960	Kinetes	Perforin-like protein (PLP5)	54.43	BBOV_II001970	48%
BBBOND_0103080	Kinetes	Perforin-like protein (PLP2)	257.99	BBOV_II007150	62%
BBBOND_0202000	Kinetes	Perforin-like protein (PLP4)	42.47	BBOV_II002020	47%
BBBOND_0301750	Kinetes	Perforin-like protein (PLP3)	177.80	no *B. bovis* ortholog	N/A
BBBOND_0301590	Kinetes	Perforin-like protein (PLP6)	129.25	BBOV_III000320	49%
BBBOND_0301700	Kinetes	Perforin-like protein (PLP7)	49.77	BBOV_III000412	48%
BBBOND_0301690	Kinetes	Perforin-like protein (PLP8)	88.42	BBOV_III000410	65%
BBBOND_0402480	Blood-stages	Perforin-like protein (PLP1)	1.65	BBOV_IV001370	58%
BBBOND_0400170	Kinetes	Aspartyl protease (ASP)	15.52	BBOV_IV007890	69%
BBBOND_0108020	Blood-stages	Aspartyl protease (ASP)	8.64	BBOV_IV010360	49%
BBBOND_0403010	Blood-stages	Fructose-1,6-bisphosphate aldolase	1.70	BBOV_IV000790	91%
BBBOND_0310920	Blood-stages	Inner membrane protein	3.45	no *B. bovi*s ortholog	N/A
BBBOND_0109200	Blood-stages	Apical membrane antigen 1	53.64	BBOV_IV011230	55%
BBBOND_0102310	Blood-stages	Papain	33.08	BBOV_III010070	49%
BBBOND_0107368	Blood-stages	Rhoptry-associated proteins (RAP-1c)	39	BBOV_IV009860 (RAP-1) BBOV_IV009870 (RAP-1)	31% 31%
BBBOND_0107940	Blood-stages	RAP-1 related antigen (RRA)	56.69	BBOV_IV010280	39%
BBBOND_0205970	Blood-stages	45 kilodaltons glycoprotein (GP-45)	25.7	no *B. bovis* ortholog	N/A
BBBOND_0206730	Kinetes	Kinete stage-specific protein	20,159.79	BBOV_I002220	25%

### Microneme

Host cell invasion/egress is largely regulated by the secretion of proteins from specialized organelles, such as micronemes. Many studies have identified the most common post-translational modification to be phosphorylation by protein kinases (PK) and protein phosphatases (PP). Calcium-dependent phosphorylation, driven by a unique family of calcium-dependent protein kinases (CDPKs), plays an essential role in regulation of the protein secretion and increase in the parasite’s cytosolic calcium concentration (Frénal and Soldati-Favre, 2013; Yang and Arrizabalaga, 2017). Calcium-dependent proteins are important for motility, cell invasion, and egress of Apicomplexa parasites (Chakraborty et al., 2017; Dubois and Soldati-Favre, 2019). In *B. bigemina* kinetes, CDPK4 and CDPK were upregulated (**Table 7**, **Table 8**). In *B. bovis* kinetes, the expression of CDPK4 (BBOV_IV003210, 76-fold) and CDPK (BBOV_II007640, 3-fold) were also upregulated (Ueti et al., 2021). Protein phosphatases are characterized based on target amino acid: serine/threonine, tyrosine, or dual specificity (acting on the three residues) (Yang and Arrizabalaga, 2017). In *B. bigemina*, the targets of all three PP identified (PP, PP2A and PP2C) were serine/threonine (**Table 8**). In addition to cell motility, PP2A is also involved in other biological processes such as proliferation, apoptotic cell death, and cell cycle control (Lillo et al., 2014). In *B. bovis* kinetes, PP2C (BBOV_I000710, 7.7-fold) was upregulated (Ueti et al., 2021). In *B. bigemina* kinetes, a single PPKL serine/threonine phosphatase was found (**Table 8**), corroborating with Yang and Arrizabalaga (2017) who found that four apicomplexan parasites (*Cryptosporidium parvum*, *B. bovis*, *P. falciparum*, and *T. gondii*) have only one PPKL.

### Perforin-like proteins

Perforins form pores in the PM of the invertebrate or mammalian host cells facilitating the parasite’s egress process or assisting in traversing epithelial barriers during tissue passage (Sassmannshausen et al., 2020). In a recent study, 38 members of the perforin-like protein (PLP) gene family were identified in *B. bovis*, *B. bigemina*, *B. ovata*, *B. divergens*, *B. microti* and *B. canis* (Paoletta et al., 2021). However, unlike the data reported by Paoletta et al. (2021), during the *B. bovis* genome re-annotation process, it was established that gene BBOV_III000410 was split in two (BBOV_III000410 and BBOV_III000412), totaling seven PLPs identified in *B. bovis* (Ueti et al., 2021). Also, by bioinformatics tools, 60% of the *Babesia* PLP gene family have a signal peptide, suggesting that these proteins are secreted or reside in the lumen of the organelles (Paoletta et al., 2021). In *B. bigemina* RNA-seq, all PLPs2-8 genes were differentially upregulated in kinetes, with a 42- to 258-fold increase, and only PLP1 in *B. bigemina* blood-stages with a 1.65-fold increase (**Table 8**). As previously described, PLPs were upregulated in *B. bovis* kinetes with 10 to 870-fold increase, and only one PLP in blood-stages with 62-fold increase (Ueti et al., 2021). In addition, four more genes containing membrane attack complex/perforin (MACPF) domains (BBOV_II006750, BBOV_III000412, BBOV_III000414 with 17 to 654-fold increase) were identified in *B. bovis* kinetes (Ueti et al., 2021).

### Bridging proteins

For binding of the actin-myosin motor to occur, bridging proteins are required. Two bridging proteins have been identified in Apicomplexa (Boucher and Bosch, 2015). Both bridging proteins were found in *B. bigemina* fructose-1,6-bisphosphate aldolase, and glyceraldehyde 3-phosphate dehydrogenase (GAPDH) (BBBOND_0403010 and BBBOND_0202520) (**Table 8**). However, the change of GAPDH expression was not significant between blood-stages and kinetes. In *B. bovis*, the expression of orthologous aldolase BBOV_IV000790 was not significant in kinetes or blood-stages, and GAPDH BBOV_II002540 had a 3.7-fold increase in blood-stages (Ueti et al., 2021).

### Thrombospondin-related anonymous proteins

A previously study demonstrated that TRAP proteins were associated with parasite invasion (Gaffar et al., 2004). In *B. bigemina*, we identified three TRAP genes. TRAP1 was overexpressed in kinetes whereas two TRAPs were upregulated in *B. bigemina* blood-stages (**Table 8**). In *B. bovis*, a TRAP-family protein was found on the apical side of merozoites (blood-stage) and is involved in host cell invasion (Gaffar et al., 2004). In a previous *B. bovis* RNA-seq, TRAP1 (BBOV_II002650) was also found to be upregulated in kinetes with 1,750-fold increase, while in blood-stages, TRAP2 (BBOV_II002890), TRAP3 (BBOV_II002630), and TRAP4 (BBOV_II002870) by 11-, 4- and 1.5-fold change, respectively (Ueti et al., 2021). A previous study demonstrated that TRAP1 protein was expressed only by *B. bovis* kinetes. In contrast, TRAP2, 3, and 4 proteins were expressed on the surface of *B. bovis* blood and sexual stages (Masterson et al., 2022).

### Aspartyl protease 3

The secretory protein aspartyl protease 3 (ASP3) is associated with the rhoptry discharge during invasion of *T. gondii* and with the lysis of the host cell membrane during egress (Dogga et al., 2017). An anti-malarial compound (49c) based on a hydroxyethylamine scaffold interrupts the lytic cycle of *T. gondii* tachyzoites by targeting ASP3, and blocks invasion, egress and rhoptry discharge (Dogga et al., 2017). In *B. bigemina*, two ASPs were differentially upregulated in kinetes and blood-stages (**Table 8**). In *B. bovis* RNA-seq, ASPs (BBOV_IV007890, 35-fold and BBOV_IV009660, 9.8-fold) were found in kinetes, and in blood-stages (BBOV_IV010360, 21.8-fold and BBOV_III003510, 2.6-fold).

### ROM proteases

ROM proteases contribute to processing of adhesins involved in attachment, invasion, intracellular replication, phagocytosis, and immune evasion, placing them at the vertex of host–parasite interactions (Sibley, 2013). ROM may be involved in releasing the parasite into the parasitophorous vacuole and RBC invasion by *Babesia* (Li et al., 2012; Suarez et al., 2019). In *B. bovis*, the parasitophorous membrane dissolves in a few minutes after merozoite invasion into the RBC (Asada et al., 2012). In *P. falciparum*, ROM4 function is related to cleavage of the adhesins of the cell surface during the invasion of RBC (O’Donnell et al., 2006). *Babesia bigemina* and *B. bovis* have three to five ROM4, and one ROM6, ROM7 and ROM8 (Gallenti et al., 2021; Gallenti et al., 2022). In *B. bigemina* RNA-seq, kinetes and blood-stages expressed genes encoding ROM proteases (**Table 8**). In addition, ROM6 (BBBOND_0208010) was also found in the RNA-seq of *B. bigemina*, however, it was not significant in the groups analyzed. In *B. bovis* RNA-seq, all ROM4 proteins were found in blood-stages (BBOV_II005940 [ROM4.4], BBOV_II005930 [ROM4.3], and BBOV_II005950 [ROM4.5], 150-, 14- and 6-fold change) and in kinetes (BBOV_II006100 [ROM4.1] and BBOV_II006070 [ROM4.2], 13,286.23 and 3,000-fold). In addition, ROM6 (BBOV_I003700, 1.54-fold in kinetes), ROM7 (BBOV_III000530, 2.67-fold in blood-stages) and ROM8 (BBOV_IV005790, 1.5-fold in blood-stages) were also upregulated (Ueti et al., 2021).

### Rhoptry-associated proteins

Rhoptry-associated proteins are conserved in several species of *Babesia*, play an important functional role in parasite invasion and have been studied as a component in a vaccine against bovine babesiosis (Brown and Palmer, 1999). Monoclonal antibodies against *B. bigemina* and *B. bovis* RAP-1 inhibit parasite invasion (Figueroa and Buening, 1991; McElwain et al., 1991; Mosqueda et al., 2002). Like *B. bovis* (BBOV_IV009870 and BBOV_IV009860, with 122- and 35-fold increase, respectively), *B. bigemina* RAP-1 has been observed in blood-stages parasites (**Table 8**). In fact, there are important differences in the structure and organization of RAP-1 genes among these two parasites. While *B. bovis* only contains two identical RAP-1 genes, *B. bigemina* contains genes encoding for four distinct RAP-1a genes, five identical RAP-1b genes and a single copy RAP-1c gene (Suarez et al., 2003). Previous expression analysis performed on cultured blood-stages of the parasites revealed that only RAP-1a genes express RAP1a proteins, but the RAP-1b and RAP-1c genes generate low levels of transcripts, with no significant amounts of detectable proteins (Suarez et al., 2003). A RAP-1 related antigen (RRA) gene contributes to erythrocyte invasion and/or egression by *B. bovis*, and overall, to the survival of the parasite during infection (Suarez et al., 2011) and was found in *B. bigemina* (**Table 8**) and in *B. bovis* blood-stages (BBOV_IV010280, 18-fold increase) (Ueti et al., 2021).

### Glycoprotein 45

The 45 kilodaltons glycoprotein (GP-45) is exposed on the surface of the *B. bigemina* merozoite, is highly immunogenic and it is believed to play a role in the invasion of erythrocytes (Mercado-Uriostegui et al., 2022). Nucleotide and amino acids sequences of GP-45 of 14 field isolates and strains revealed high percentage of similarity. In addition, sera from cattle naturally infected with *B. bigemina* contain antibodies that bind to specific peptides of GP-45 (Mercado-Uriostegui et al., 2022). In blood-stages of *B. bigemina*, the gene corresponding to GP-45 was upregulated (**Table 8**). *Babesia bigemina* GP-45 appears to be present as a single-copy gene (Fisher et al., 2001). In addition, GP-45 lacks introns and, unlike the tandem arrangement of *B. bovis* MSA-2 genes, GP45 is flanked by unrelated ORFs, similar to *B. bovis* MSA-1 (Suarez et al., 2000; Fisher et al., 2001). In *B. bovis*, all variable MSA (MSA1, MSA-2a1, MSA-2a2, MSA-2b and MSA-2c, with 86- to 170-fold) were found in blood-stages (Ueti et al., 2021).

### Kinete-specific protein

The KSP gene that has been implicated to be important in transovarial transmission (Johnson et al., 2017; Bohaliga et al., 2019) was highly transcribed and specifically expressed in *B. bigemina* kinetes (**Table 2**, **Table 8**). This gene is homologous to the *B. bovis* KSP protein, sharing 25% amino acid identity. The fold change of both *B. bigemina* and *B. bovis* KSPs was similar (**Table 2**). In the *B. bovis* genome, a GPI anchorage site was identified in KSP, suggesting that the protein may be anchored on the parasite’s surface, and may play a role in invading tick ovary epithelial cells (Rodriguez et al., 2014; Johnson et al., 2017). So far, it is not known if other protozoan parasites share this protein (Johnson et al., 2017; Bohaliga et al., 2019). In fact, this gene does not appear in *Theileria* spp. or *B. microti* (**Supplementary Table 3**), perhaps because these parasites are not transovarially transmitted to the tick’s offspring (Jalovecka et al., 2018).

### Genes upregulated by members of *Babesia sensu stricto* not found in *Theileria* spp. and *Babesia microti*

*Babesia sensu stricto*, such as *B. bigemina* and *B. bovis*, refers to protozoan parasites that are transovarially transmitted by the tick vectors. In contrast, *Babesia sensu lato*, such as *B. microti*, or *Theileria* are not transovarially transmitted by the vector (Schreeg et al., 2016; Jalovecka et al., 2018; Jalovecka et al., 2019). Discovering key molecules involved in *Babesia sensu stricto* transmission may lead to the development of interfering strategies to block the biological transmission of these parasites (Suarez et al., 2019). To identify genes unique to *Babesia sensu stricto* that are potentially involved in vertical transmission, all 906 genes upregulated in kinetes of *B. bigemina* were compared by BLASTp with *B. bovis*, *Theileria* spp. and *B. microti* genomes available in NCBI dataset. Of the genes upregulated in *B. bigemina* and *B. bovis* kinetes, 44 were not found in *Theileria* spp. and *B. microti* genomes (**Supplementary Table 3**). The genes listed in **Supplementary Table 3** include KSP, membrane proteins, and proteins of unknown function. Some of these genes may encode proteins that have a function in kinete invasion into ovaries and transovarial transmission.

### Validation of transcription levels by qPCR

To validate the RNA seq, four genes from kinete (TRAP1, ROM4, Der-1, and KSP) and four blood-stage genes (SBP4, CCp2, PFruc, and Ribok) were selected based on their magnitude of transcription. The similar profile of two reference genes (MAPK and ProtA) demonstrated a consistent pattern of the differential gene expression between kinete and blood-stage parasites (**Supplementary Figure 2**).

## Conclusion

Omic studies that include cattle *Babesia* focus on analyzing genes of *Babesia* strains with distinct virulence, or between free merozoites *versus* intraerythrocytic stages (Pedroni et al., 2013; González et al., 2019). In contrast, our study compared genes differentially expressed by two different stages of the developmental cycle of *B. bigemina*: kinetes in the tick vector and blood-stages in bovine RBC. This approach revealed potential essential genes involved in parasite development within the tick and mammalian hosts.

Knowledge and understanding of the changes in gene expression patterns that occur as a result of mammalian-tick transitions is crucial for identifying candidates for development of efficient control strategies for bovine babesiosis. The results herein revealed important genetic markers to develop strategies to control the spread of bovine babesiosis caused by *B. bigemina* and *B. bovis*. Specifically, genes identified herein that are upregulated in both *B. bigemina* and *B. bovis* kinete could be used to determine critical genes or proteins to prevent tick infection and, consequently disrupt the transmission of the pathogen *via* tick vectors.

In this study, we provided a robust panel of expressed genes for *B. bigemina* by two distinct developmental stages and revealed potential markers of transovarial transmission. However, this study is based on differential expression gene analysis, and proteomic and functional studies will also be needed to strengthen our understanding of the biology of these parasites. We propose that the results generated in this study can serve as a basis for other research associated with the improvement of diagnostics, drugs, and effective vaccine development against bovine babesiosis.

## Data availability statement

The datasets presented in this study can be found in online repositories. The names of the repository/repositories and accession number(s) can be found in the article/**Supplementary Material**.

## Ethics statement

The animal study was reviewed and approved by the Institutional Animal Care and Use Committee of the University of Idaho, Moscow, ID, USA.

## Author contributions

MU and WJ conceived and supervised the project. JC-P drafted manuscript. JC-P, WJ, LK, and NT prepared figures and tables. KR, WJ and MU performed the experiments. JC-P, PS, WJ, LK, and MU analyzed the data. WJ, LK, CS, PS, HM, NT, KB and MU wrote and edited the manuscript. All authors contributed to the article and approved the submitted version.
