# Supplementary material for: Comparison of high throughput RNA sequences between Babesia bigemina and Babesia bovis revealed consistent differential gene expression that is required for the Babesia life cycle in the vertebrate and invertebrate hosts
Source: Front Cell Infect Microbiol. 2022 Dec 19;12:1093338. doi: 10.3389/fcimb.2022.1093338 (PMC9806345; doi:10.3389/fcimb.2022.1093338)
Supplement: Supplementary file 2 [file Presentation_1.pdf]

Supplementary Figure 1: RT-PCR spanning two *B. bigemina* GCC2/GCC3 gene models.

```
8310       TAAGCGTTACGTGTTATTGCTCAGCTGGCCGTGTGTTGGGAGTTTTTGTTCTTTAATCGA    60
PCR        TAAGCGTTACGTGTTATTGCTCAGCTGGCCGTGTGTTGGGAGTTTTTGTTCTTTAATCGA    60
8320       ------------------------------------------------------------    0
BBBOND     TAAGCGTTACGTGTTATTGCTCAGCTGGCCGTGTGTTGGGAGTTTTTGTTCTTTAATCGA    60


8310       AGCTAAAGAGAGCGTCGGACTTGTTTCCAATATTATATTGCAGGGTTCGTTTACCTTTTA    120
PCR        AGCTAAAGAGAGCATCGGACTTGTTTC---------------------------------    87
8320       ------------------------------------------------------------    0
BBBOND     AGCTAAAGAGAGCGTCGGACTTGTTTCCAATATTATATTGCAGGGTTCGTTTACCTTTTA    120


8310       G-----------------------------------------------------------    121
PCR        ------------------------------------------------------------    87
8320       ------------------------------------------------------------    0
BBBOND     GAATACATACTGTTTTATACATGTACCTCTGTGTCATCTGCTAATGTATACATGGCCTGC    180


8310       ------------------------------------------------------------    121
PCR        ------------------------------------------------------------    87
8320       ------------------------------------------------------------    0
BBBOND     ATATTGCTGAATGGCCACATCAATGCATTTGTAATGAATCAATTTTGCATAGCGGTAGTT    240


8310       ------------------------------------------------------------    121
PCR        -----------------------------------CAATATTATATTGCAGGGCCCAGTG    112
8320       ------------------------------------------------------------    0
BBBOND     TCATACGTAACTACATACTCTACCTGATGACGCATTTTAAAACTTTGCTTAGGTCCAGTG    300


8310       ------------------------------------------------------------    121
PCR        CAACCGGTGAAAAGGATCTGCACGGTTGGTGCATTATGCCGATTTTCGCTTCCAACTGTG    172
8320       ------------------------------------------------------------    0
BBBOND     CAACCGGTGAAAAGGATCTGCACGGTTGGTGCATTATGCCGATTTTCGCTTCCAACTGTG    360


8310       ------------------------------------------------------------    121
PCR        AACATGCCTGAAAAGCAGATTCCCCCTGTGATCATTTCCAAAAAATGCACGCCAGGTAGA    232
8320       ---ATGCCTGAAAAGCGGATTCCCCCTGTGATCATTTCCAAAAAGTGCACGCCAGATACA    57
BBBOND     AACATGCCTGAAAAGCAGATTCCCCCTGTGATCATTTCCAAAAAATGCACGCCAGGTAGA    420
```

Sequences encoding GCC2/GCC3 domain containing proteins of *B. bigemina* was obtained by conventional PCR using forward primer from sequence BBBOND_0208310 (5'GCGATGACGGAAGTCAGAAT3') and reverse primer from BBBOND_0208320 (5'ATCCGTATGTTCCACGAAGG3'). PCR were conducted in a 20 µL reaction volume using 10 µL of JumpStart RedTaq Mix (Sigma Aldrich, USA), 1 µL of each primer ([10 µM])**,** 7 µL of nuclease free water and 1 µL of *B. bigemina* kinete cDNA as template. The cDNA was prepared using SuperScript III^{TM} cDNA Synthesis kit (ThermoFisher Scientific, USA) according to the manufacturer's instructions. The PCR conditions were: 95 ^{o}C for 3 min followed by 35 cycles of 95 ^{o}C for 30 sec, 60 ^{o}C for 30 sec, 72 ^{o}C for 30 sec and a final elongation step of 72 ^{o}C for 5 min. The PCR product was treated by ExoSAP-IT Express reagent (Affymetrix, USB, USA) according to the manufacturer's instructions and sequenced in both directions.
