# Supplementary material for: Comparison of high throughput RNA sequences between Babesia bigemina and Babesia bovis revealed consistent differential gene expression that is required for the Babesia life cycle in the vertebrate and invertebrate hosts
Source: Front Cell Infect Microbiol. 2022 Dec 19;12:1093338. doi: 10.3389/fcimb.2022.1093338 (PMC9806345; doi:10.3389/fcimb.2022.1093338)

Supplementary Figure 2: Replicate samples generated from a bovine infected with *B. bigemina* and kinetes were used to validate the RNA-seq by quantitative PCR. Normalization was performed using two housekeeping genes A) MAPK and B) ProtA. Red bars indicate *B. bigemina* blood-stages and blue bars indicate kinetes.

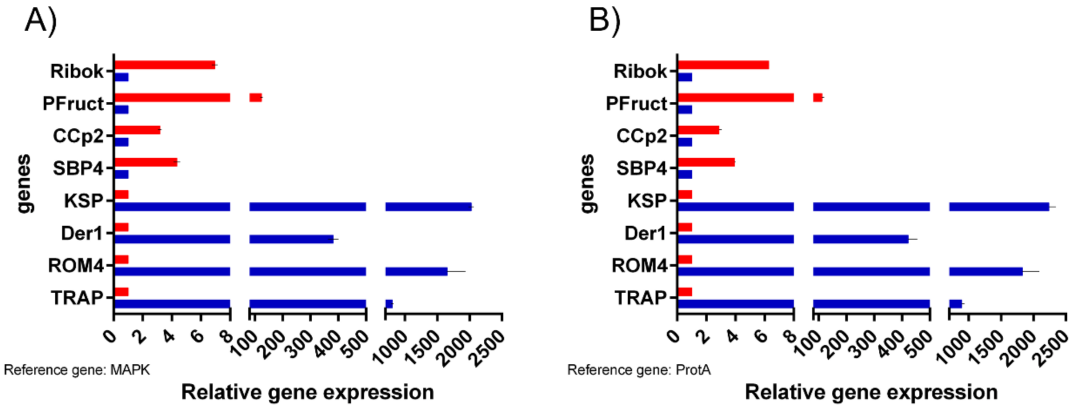

Supplement: Supplementary file 3 [file Presentation_2.pdf]
