# Supplementary material for: Comparison of high throughput RNA sequences between Babesia bigemina and Babesia bovis revealed consistent differential gene expression that is required for the Babesia life cycle in the vertebrate and invertebrate hosts
Source: Front Cell Infect Microbiol. 2022 Dec 19;12:1093338. doi: 10.3389/fcimb.2022.1093338 (PMC9806345; doi:10.3389/fcimb.2022.1093338)
Supplement: Supplementary file 4 [file Table_1.docx]

Supplementary Table 1: Primers used in this study for validation of RNA-seq by quantitative PCR.

| **Gene** | **Name** | **Primer-Fw (5'-3')** | **Primer-Rv (5'-3')** | **Amplicon (bp)** |
| --- | --- | --- | --- | --- |
| BBBOND_0202740 | Thrombospondin-related anonymous protein (TRAP) | GAGGAAAGGCTGAAGCAAGA | AGTTGTCGCTCCCTCTTCAT | **108** |
| **BBBOND_0104040** | **rhomboid 4 (Rom4)** | CGAAGGAGATGAGGGGTAAA | **GCTTTTCTAGCGGGTTGATG** | **108** |
| BBBOND_0403740 | Der1-like family (Der1) | TGGCCTTGTCGAGTATTTCC | CTCGCCACGTTTCACTCTTT | **101** |
| BBBOND_0206730 | kinete stage-specific protein (KSP) | GCTGATGGCACCAAAGAGTT | CGAACGCATCCTTAACCATC | **109** |
| BBBOND_0209400 | spherical body protein 4 (SBP4) | ACGACGTCAACGACAACAAG | TTGTCGGAGTAGAAGCACGA | **100** |
| BBBOND_0203950 | LCCL domain-containing protein (CCp2) | ACAACAAGTGCAACGCTGAG | TTGACCACGAACTCCACCTT | **109** |
| BBBOND_0100430 | Probable fructokinase (PFruct) | GCTATCGGCTTGTACGTGAA | TCTCCAATGCTGGAGAAAGC | **104** |
| BBBOND_0100530 | Ribokinase - like superfamily protein (Ribok) | TCGACACTAAGACCCCTACTGA | GTCGGCCAAATCATCCTTAC | **110** |
| BBBOND_0209200 | Mitogen-activated protein kinase (MAPK) | ACTTCCAGCGCAAGAAGATG | TCGCGGAGGTGTAGTTTTCT | **117** |
| BBBOND_0104190 | Proteosome A (ProtA) | GGATACATCACGGCGTTTCT | AGCTGTTGTCGAGGTCGATT | **112** |
