# Supplementary material for: Comparison of high throughput RNA sequences between Babesia bigemina and Babesia bovis revealed consistent differential gene expression that is required for the Babesia life cycle in the vertebrate and invertebrate hosts
Source: Front Cell Infect Microbiol. 2022 Dec 19;12:1093338. doi: 10.3389/fcimb.2022.1093338 (PMC9806345; doi:10.3389/fcimb.2022.1093338)
Supplement: Supplementary file 5 [file Table_2.docx]

Supplementary Table 2: Sequence mapping depth and quality of *B. bigemina* RNA from calf infected blood and tick hemolymph.

| **Sample** | **Mapped (Million reads)** | **Mapped paired (Million reads)** | **Fragment length mean (bp)** | **rRNA rate** |
| --- | --- | --- | --- | --- |
| Calf 1 | **27447279** | **10074143** | **125** | **.087** |
| Calf 2 | **38789110** | **14189666** | **126** | **.080** |
| Calf 3 | **26081321** | **9532935** | **124** | **.096** |
| Tick 1 | **5233803** | **1755170** | **126** | **.024** |
| Tick 2 | **14289691** | **4726417** | **127** | **.020** |
| Tick 3 | **7646465** | **2509335** | **125** | **.015** |

Approximately 60 million total reads per sample were obtained.
