# Supplementary material for: Comparison of high throughput RNA sequences between Babesia bigemina and Babesia bovis revealed consistent differential gene expression that is required for the Babesia life cycle in the vertebrate and invertebrate hosts
Source: Front Cell Infect Microbiol. 2022 Dec 19;12:1093338. doi: 10.3389/fcimb.2022.1093338 (PMC9806345; doi:10.3389/fcimb.2022.1093338)
Supplement: Supplementary file 6 [file Table_3.docx]

Supplementary Table 3: Protein databases search by species *B. bovis*, *Theileria* spp., and *B. microti* using a *B. bigemina* RNAseq kinetes protein query, by Blastp platform. *B. bigemina* proteins found in *B. bovis*, but with no corresponding genes in *Theileria* spp. and *B. microti*, are detailed.

| **GeneID** | ***B. bigemina***  **Annotation** | **Fold increase in *B. bigemina* kinetes** | **Comments** | ***B. bovis* (T2Bo)**  **Annotation** | **Query Cover (%)/ Identity to *B. bovis* (protein)(%)** | **E value** | | **Blastp #** | **Fold increase in *B. bovis* kinetes** | ***Theileria* spp.** | ***B. microti*** |
| --- | --- | --- | --- | --- | --- | --- | --- | --- | --- | --- | --- |
| BBBOND_0206730 | Kinete Specific protein | 20159.79 | Location: chromosome II  612 aa protein | Protein of unknown function  BBOV_I002220 | 96/22.89 | 6.00E-40 | | XP_001608872.1 | 19316.40 | N.D. | N.D. |
| BBBOND_0108780 | Protein of unknown function | 17454.75 | Location: chromosome I  174 aa protein | Protein of unknown function  BBOV_IV011035 | 63/35.96 | 1.00E-15 | | KAG6439940.1 | 18702.84 | N.D. | N.D. |
| BBBOND_0402020 | Protein of unknown function | 6244.72 | 2 Large tegument domains  Location: chromosome IV  1477 aa protein | membrane protein  BBOV_III005970 | 35/33.64 | 7.00E-16 | | XP_001611726.1 | 15766.71 | N.D. | N.D. |
| BBBOND_0405500 | Protein of unknown function | 1354.80 | Vesicle coat complex COPII domain  Location: chromosome V  147 aa protein | Protein of unknown function  BBOV_IV001565 | 100/53.33 | 4.00E-48 | | KAG6439894.1 | 351.26 | N.D. | N.D. |
| BBBOND_0103640 | membrane protein | 972.84 | Location: chromosome I  256 aa protein | membrane protein  BBOV_II006530 | 92/48.75 | 7.00E-26 | | XP_001610171.1 | 209.18 | N.D. | N.D. |
| BBBOND_0310060 | Protein of unknown function | 790.02 | Location: chromosome III  253 aa protein | Protein of unknown function  BBOV_III004770 | 33/28.74 | 1.00E-05 | | XP_001611608.1 | 55.83 | N.D. | N.D. |
| BBBOND_0203830 | Protein of unknown function | 499.94 | Location: chromosome II  255 aa protein | Protein of unknown function  BBOV_II003620 | 100/32.45 | 1.00E-25 | | EDO06317.2 | 14000.99 | N.D. | N.D. |
| BBBOND_0400470 | Protein of unknown function | 276.79 | DNA topoisomerase 2-like domain  Location: chromosome IV  769 aa protein | Protein of unknown function  BBOV_IV007620 | 6/37.93 | 0.004 | | XP_001610684.1 | 101.92 | N.D. | N.D. |
| BBBOND_0205500 | Protein of unknown function | 210.17 | Location: chromosome II  97 aa protein | putative integral membrane protein  BBOV_I002745 | 50/40.82 | 8.00E-12 | | KAG6440194.1 | 6588.76 | N.D. | N.D. |
| BBBOND_0305710 | Protein of unknown function | 176.61 | Prolipoprotein diacylglyceryl transferase domain  Location: chromosome III  347 aa protein | Protein of unknown function  BBOV_III008230 | 40/29.29 | 9.00E-20 | | XP_001611951.1 | 31.55 | N.D. | N.D. |
| BBBOND_0312260 | Protein of unknown function | 172.12 | Chromosome segregation protein SMC domain  Location: chromosome III  539 aa protein | chromosome segregation ATPase family protein  BBOV_III008784 | 96/30.20 | 6.00E-59 | | KAG6440056.1 | 41.26 | N.D. | N.D. |
| BBBOND_0303410 | Protein of unknown function | 137.06 | Location: chromosome III  133 aa protein | Protein of unknown function  BBOV_III001935 | 90/24 | 0.025 | | KAG6440067.1 | 182.72 | N.D. | N.D. |
| BBBOND_0206160 | Protein of unknown function | 93.45 | Location: chromosome II  350 aa protein | Protein of unknown function  BBOV_I002670 | 95/37.39 | 9.00E-64 | | EDO05349.2 | 308.88 | N.D. | N.D. |
| BBBOND_0302970 | membrane protein | 62.48 | Location: chromosome III  277 aa protein | membrane protein  BBOV_III001620 | 34/28.12 | 7.00E-12 | | XP_001611297.1 | 114.03 | N.D. | N.D. |
| BBBOND_0306630 | membrane protein | 62.02 | Location: chromosome III  196 aa protein | membrane protein  BBOV_III007640 | 94/34.76 | 3.00E-29 | | XP_001611892.1 | 436.98 | N.D. | N.D. |
| BBBOND_0309780 | Protein of unknown function | 57.64 | Location: chromosome III  881 aa protein | Protein of unknown function  BBOV_III004950 | 96/45.95 | 0 | | XP_001611626.1 | 115.06 | N.D. | N.D. |
| BBBOND_0312990 | Protein of unknown function | 43.82 | SMC - Chromosome segregation ATPase  Location: chromosome III  394 aa protein | Protein of unknown function  BBOV_III009300 | 100/29.62 | 2.00E-42 | | XP_001612054.1 | 10.28 | N.D. | N.D. |
| BBBOND_0405830 | Protein of unknown function | 28.59 | Location: chromosome V  818 aa protein | Protein of unknown function  BBOV_I004880 | 88/36.33 | 3.00E-121 | | XP_001609137.1 | 6.13 | N.D. | N.D. |
| BBBOND_0204710 | Protein of unknown function | 26.49 | ribonuclease E, Ring-infected erythrocyte surface antigen and Midasin domains  Location: chromosome II  1445 aa protein | Protein of unknown function  BBOV_II004370 | 18/36.16 | 2.00E-48 | | EDO06392.2 | 906.75 | N.D. | N.D. |
| BBBOND_0305990 | Protein of unknown function | 21.54 | Location: chromosome III  727 aa protein | Protein of unknown function  BBOV_III004410 | 100/49.14 | 3.00E-30 | | XP_001611572.1 | 68.36 | N.D. | N.D. |
| BBBOND_0102380 | Protein of unknown function | 19.77 | Location: chromosome I  185 aa protein | Protein of unknown function  BBOV_II002940 | 98/81.45 | 1.00E-89 | | XP_001609817.1 | 1.73 | N.D. | N.D. |
| BBBOND_0310670 | Protein of unknown function | 18.71 | Location: chromosome III  156 aa protein | Protein of unknown function  BBOV_III004385 | 100/62.82 | 1.00E-63 | | KAG6440003.1 | 11.64 | N.D. | N.D. |
| BBBOND_0305480 | Protein of unknown function | 17.95 | Location: chromosome III  139 aa protein | Protein of unknown function  BBOV_III008405 | 71/47 | 3.00E-30 | | KAG6440074.1 | 48.88 | N.D. | N.D. |
| BBBOND_0203600 | Protein of unknown function | 17.35 | Location: chromosome II  375 aa protein | Protein of unknown function  BBOV_II003444 | 96/48.16 | 1.00E-112 | | KAG6440154.1 | 5.62 | N.D. | N.D. |
| BBBOND_0104140 | Protein of unknown function | 15.78 | Location: chromosome I  224 aa protein | Protein of unknown function (putative integral membrane protein)  BBOV_II006020 | 86/48.98 | 7.00E-44 | | XP_001610120.1 | 7.05 | N.D. | N.D. |
| BBBOND_0403530 | Protein of unknown function | 12.39 | Location: chromosome IV  133 aa protein | ABC1_ADCK3-like family protein  BBOV_IV000470 | 89/33.61 | 5.00E-12 | | EDO05647.2 | 1.77 | N.D. | N.D. |
| BBBOND_0313000 | Protein of unknown function | 12.33 | Smc - Chromosome segregation ATPase domain  Location: chromosome III  317 aa protein | Protein of unknown function  BBOV_III009300 | 98/36.31 | 2.00E-30 | | EDO08486.2 | 10.28 | N.D. | N.D. |
| BBBOND_0403130 | Protein of unknown function | 11.76 | Location: chromosome IV  1536 aa protein | Protein of unknown function  BBOV_IV000890 | 38/36.77 | 5.00E-89 | | XP_001609255.1 | 1.72 | N.D. | N.D. |
| BBBOND_0311180 | membrane protein | 11.55 | Location: chromosome III  397 aa protein | membrane protein  BBOV_III004180 | 100/25.30 | 3.00E-21 | | XP_001611549.1 | 30.26 | N.D. | N.D. |
| BBBOND_0109370 | Protein of unknown function | 11.21 | Location: chromosome I  435 aa protein | Protein of unknown function  BBOV_IV011300 | 87/25.19 | 2.00E-30 | | EDO07482.2 | 175.74 |  | N.D. |
| BBBOND_0110080 | Protein of unknown function | 10.34 | Location: chromosome I  204 aa protein | Protein of unknown function  BBOV_IV011800 | 84/38.29 | 9.00E-25 | | XP_001611100.1 | 11.16 | N.D. | N.D. |
| BBBOND_0405600 | Protein of unknown function | 9.54 | Alpha-crystallin domain (ACD) of alpha-crystallin-type small(s) heat shock proteins (Hsps)  Location: chromosome V  313 aa protein | Hsp20/alpha crystallin family protein  BBOV_IV001650 | 89/34.04 | 1.00E-37 | | EDO05762.2 | 1.67 | N.D. | N.D. |
| BBBOND_0101490 | Protein of unknown function | 9.17 | ISP3 C-terminal domain  Location: chromosome I  174 aa protein | Protein of unknown function  BBOV_III010670 | 86/65.56 | 3.00E-69 | | XP_001612187.1 | 0.41 | N.D. | N.D. |
| BBBOND_0303220 | Protein of unknown function | 6.00 | Location: chromosome III  427 aa protein | Protein of unknown function  BBOV_III001800 | 80/37.39 | 1.00E-78 | | EDO07747.2 | 8.72 | N.D. | N.D. |
| BBBOND_0307280 | Protein of unknown function | 5.75 | Location: chromosome III  72 aa protein | putative integral membrane protein  BBOV_III006834 | 98/47.89 | 5.00E-19 | | KAG6440097.1 | 6.46 | N.D. | N.D. |
| BBBOND_0304910 | Protein of unknown function | 4.75 | Location: chromosome III  177 aa protein | Protein of unknown function  BBOV_III003005 | 100/46.70 | 1.00E-46 | | KAG6439991.1 | 53.86 | N.D. | N.D. |
| BBBOND_0204110 | Protein of unknown function | 3.91 | Location: chromosome II  385 aa protein | Protein of unknown function  BBOV_II003850 | 100/47.15 | 6.00E-98 | XP_001609908.1 | | 0.42 | N.D. | N.D. |
| BBBOND_0310130 | Protein of unknown function | 3.57 | Location: chromosome III  74 aa protein | putative integral membrane protein  BBOV_III004710 | 97/34.72 | 1.00E-06 | EDO08034.2 | | 18.70 | N.D. | N.D. |
| BBBOND_0102620 | Protein of unknown function | 3.49 | SMC - Chromosome segregation ATPase domain  Location: chromosome I  293 aa protein | Protein of unknown function  BBOV_II007660 | 91/39.55 | 5.00E-32 | XP_001610284.1 | | 3.13 | N.D. | N.D. |
| BBBOND_0300980 | Protein of unknown function | 3.27 | Location: chromosome III  274 aa protein | Protein of unknown function  BBOV_I000620 | 81/27.35 | 3.00E-17 | EDO05156.2 | | 1.33 | N.D. | N.D. |
| BBBOND_0207300 | Protein of unknown function | 2.59 | Location: chromosome II  155 aa protein | Protein of unknown function  BBOV_I001730 | 73/43.59 | 6.00E-24 | XP_001608825.1 | | 4.07 | N.D. | N.D. |
| BBBOND_0202970 | Protein of unknown function | 2.58 | Location: chromosome II  830 aa protein | Protein of unknown function  BBOV_III003690 | 98/36.17 | 2.00E-142 | EDO07933.2 | | 1.63 | N.D. | N.D. |
| BBBOND_0110340 | Protein of unknown function | 2.54 | Location: chromosome I  231 aa protein | Protein of unknown function  BBOV_IV011920 | 41/31.31 | 5.00E-04 | XP_001611112.1 | | 7.32 | N.D. | N.D. |
| BBBOND_0206940 | Protein of unknown function | 2.04 | Location: chromosome II  333 aa protein | Protein of unknown function  BBOV_I002040 | 93/6.23 | 3.00E-50 | XP_001608855.1 | | 2.49 | N.D. | N.D. |

N.D. = not detected
